# Actin capping protein regulates postsynaptic spine development through CPI-motif interactions

**DOI:** 10.3389/fnmol.2022.1020949

**Published:** 2022-09-29

**Authors:** Kenneth R. Myers, Yanjie Fan, Patrick McConnell, John A. Cooper, James Q. Zheng

**Affiliations:** ^1^Department of Cell Biology, Emory University School of Medicine, Atlanta, GA, United States; ^2^Department of Biochemistry and Molecular Biophysics, Washington University in St. Louis, St. Louis, MO, United States; ^3^Department of Neurology, Emory University School of Medicine, Atlanta, GA, United States; ^4^Center for Neurodegenerative Diseases, Emory University School of Medicine, Atlanta, GA, United States

**Keywords:** synapse, CapZ, Twinfilin, cytoskeleton, dendritic spine, postsynaptic density, Shank

## Abstract

Dendritic spines are small actin-rich protrusions essential for the formation of functional circuits in the mammalian brain. During development, spines begin as dynamic filopodia-like protrusions that are then replaced by relatively stable spines containing an expanded head. Remodeling of the actin cytoskeleton plays a key role in the formation and modification of spine morphology, however many of the underlying regulatory mechanisms remain unclear. Capping protein (CP) is a major actin regulating protein that caps the barbed ends of actin filaments, and promotes the formation of dense branched actin networks. Knockdown of CP impairs the formation of mature spines, leading to an increase in the number of filopodia-like protrusions and defects in synaptic transmission. Here, we show that CP promotes the stabilization of dendritic protrusions, leading to the formation of stable mature spines. However, the localization and function of CP in dendritic spines requires interactions with proteins containing a capping protein interaction (CPI) motif. We found that the CPI motif-containing protein Twinfilin-1 (Twf1) also localizes to spines where it plays a role in CP spine enrichment. The knockdown of Twf1 leads to an increase in the density of filopodia-like protrusions and a decrease in the stability of dendritic protrusions, similar to CP knockdown. Finally, we show that CP directly interacts with Shank and regulates its spine accumulation. These results suggest that spatiotemporal regulation of CP in spines not only controls the actin dynamics underlying the formation of stable postsynaptic spine structures, but also plays an important role in the assembly of the postsynaptic apparatus underlying synaptic function.

## Introduction

During development, dendritic spines are generally thought to form as thin filopodia-like protrusions that arise from the dendritic shaft and stabilize upon contact with axons (Marrs et al., 2001; Yoshihara et al., 2009; Khanal and Hotulainen, 2021). These spines subsequently form enlarged heads, containing various postsynaptic components that are connected to the dendrite by a thin neck (Hotulainen and Hoogenraad, 2010; Chidambaram et al., 2019). The morphology of spines is tightly correlated with synaptic strength (Yuste and Bonhoeffer, 2001; Noguchi et al., 2005; Arellano et al., 2007; Araya et al., 2014). Abnormal spine shape and density contributes to numerous neurologic disorders, thus highlighting the importance of spine development for normal synaptic function (Stefen et al., 2016; Lima Caldeira et al., 2019; Nishiyama, 2019; Walker and Herskowitz, 2021). Changes in spine morphology during development or in response to synaptic activity are largely driven by remodeling of the actin cytoskeleton (Basu and Lamprecht, 2018; Borovac et al., 2018; Lamprecht, 2021). Actin is the primary cytoskeletal component in dendritic spines, and it provides the structural basis for the formation, modification, and elimination of spines during synaptic development and plasticity (Hotulainen and Hoogenraad, 2010; Lei et al., 2016; Bucher et al., 2020; Okabe, 2020).

Actin capping protein (CP, also known as CapZ), a heterodimer consisting of an α- and a β-subunit, binds to the barbed ends of actin filaments to prevent their elongation, and CP promotes the Arp2/3-mediated nucleation of short branched actin filaments essential for cell motility (Akin and Mullins, 2008; Edwards et al., 2014; Papalazarou and Machesky, 2021). CP is highly expressed in neurons, and it is concentrated in the dendritic spines of cultured hippocampal neurons (Fan et al., 2011). More specifically, CP is associated with the highly branched actin filament network within the spine head (Korobova and Svitkina, 2010). Consequently, the loss of CP in cultured hippocampal neurons results in a marked decline in spine density and a concomitant increase of filopodia-like protrusions, leading to reduced synaptic connections (Fan et al., 2011). Interestingly, the remaining spines largely exhibit aberrant morphologies, such as the presence of spine head protrusions (Fan et al., 2011). Recently, *de novo* mutations in CP were identified in children with intellectual disability and developmental delay (Huang et al., 2020; Pi et al., 2022), further highlighting the importance of CP function in brain development and function.

Capping protein interacts with a diverse set of accessory proteins that regulate its activity, including several containing capping protein interaction (CPI) motifs (Taoka et al., 2003; Falck et al., 2004; Bhattacharya et al., 2006; Hernandez-Valladares et al., 2010; Edwards et al., 2014; Stark et al., 2017). Twinfilin (Twf) contains a CPI motif, and it is a highly conserved member of the actin depolymerization factor homology (ADF-H) protein superfamily. Twf has been shown to accelerate barbed end depolymerization of actin filaments (Hilton et al., 2018). Biochemically, Twf is capable of binding to actin monomers and phosphoinositides at the plasma membrane (Palmgren et al., 2002; Hakala et al., 2018). Twf directly interacts with CP with high affinity (Poukkula et al., 2011) and *in vitro* studies indicate that Twf is able to uncap plus ends and accelerate depolymerization (Mwangangi et al., 2021; Shekhar et al., 2021). This “uncapping” action of Twf has also been demonstrated to increase CP turnover and actin dynamics in motile lamellipodia (Hakala et al., 2021). Recently, the binding of Twf to CP was shown to allosterically compete CP Arp2/3 complex myosin-I linker (CARMIL) proteins off CP, resulting in the stable capping of actin filament barbed ends (Johnston et al., 2018). These apparent pro-capping and uncapping effects of Twf, likely depend on the specific subcellular environment and/or local presence of other interacting components.

Given the importance of CP activity on dendritic spine development, we wanted to investigate the role of Twf in this highly specialized subcellular compartment. There are three mammalian isoforms of Twf: Twf-2b is expressed in heart and skeletal muscles, Twf-2a appears to be dispensable for development (Nevalainen et al., 2011), and Twinfilin-1 (Twf1) is ubiquitously expressed and found at high levels in the brain (Vartiainen et al., 2003). A previous study of the single *Drosophila* Twinfilin gene showed its involvement in a wide range of cellular events, including cell migration, axonal growth, postsynaptic localization of glutamate receptors, and presynaptic endocytosis in the neuromuscular synapse (Wang et al., 2010). A recent study has also identified Twf1 in the postsynaptic density (PSD) fraction of both human and mouse brains (Wang et al., 2020), suggesting a role for Twf1 in postsynaptic function. However, very little is otherwise known about the function of Twf1 in neurons. In this study, we report that the localization and function of CP in dendritic spines requires interactions with CPI-motif proteins, including Twf1. Furthermore, we show that the knockdown of Twf1 in cultured hippocampal neurons phenocopies CP knockdown. Both result in decreased spine density, as well as increased filopodia density and filopodia instability. Finally, we find that CP interacts with Shank to regulate its spine localization. Together, our findings support an important role for CP in regulating the F-actin structure underlying the development of postsynaptic structure and function.

## Materials and methods

### Antibodies and plasmids

To amplify green fluorescent protein (GFP) and mCherry signals for fluorescence microscopy, we used rabbit anti-GFP (A-11122; Invitrogen; 1:1,000) and rabbit anti-red fluorescent protein (RFP) (600-401-279; Rockland; 1:1000). The following antibodies were used; rabbit anti-Twinfilin-1 (1:500, generously provided by Pekka Lappalainen), rabbit anti-CP (1:500, generously provided by John Hammer), rabbit anti-6x-His (PA1-983B; Invitrogen; 1:250), anti-CPβ2 and anti-CPα1 and α2 (mAb 3F2.3 and mAb 5B12.3; Developmental Studies Hybridoma Bank; 1:100), mouse anti-pan-Shank (MABN24; Millipore; 1:500), and mouse anti-tubulin DM1A (T6199; Sigma-Aldrich; 1:5,000). GFP-CPβ2, mOrange-CPβ2, shCP-mRFP, shCP + resWT-CP [knockdown rescue (KDR)], GFP-Twf1, and mCherry-Twf1-ADF-H-domain (R96A/K98A/R267A/R269A) constructs have been described previously (Mejillano et al., 2004; Vitriol et al., 2007; Hakala et al., 2021). To generate the CP-R15A/Y79A double point mutant, we used site-directed mutagenesis to change arginine-15 and tyrosine-79 into alanines (R15A/Y79A) in both GFP-CPβ2 and shCP + resWT-CP (shCP + resR15A/Y79A-CP). To engineer shTwf1#1 and shTwf1#2, we annealed and ligated the following oligos into pSuper.neo + mCherry using the *Bgl*II/*Xho*I sites: shTwf1#1, 5′ GATCCCCGATCAGAAAGATTGAGATATTCAAGAGATATC TCAATCTTTCTGATCTTTTTC (Forward)/5′ TCGAGA AAAAGATCAGAAAGATTGAGATATCTCTTGAATATCTCA ATCTTTCTGATCGGG (Reverse); shTwf1#2, 5′ GATCCCCGGGATTAGAAGATTGATTATTCAAGAGATAAT CAATCTTCTAATCCCTTTTTC (Forward)/5′ TCGAGAAAA AGGGATTAGAAGATTGATTATCTCTTGAATAATCAATCT TCTAATCCCGGG (Reverse).

### Neuronal culture and transfection

Dissociated primary hippocampal neuron cultures were prepared as previously described (Gu et al., 2008). Briefly, 18.5 day old embryos were collected from timed-pregnant Sprague Dawley rats (Charles River Laboratories, Wilmington, MA, United States), and then hippocampi were dissected in ice-cold Hank’s Balanced Salt Solution (HBSS). Hippocampi were pooled together, trypsinized for 15 min, briefly incubated in 20% fetal bovine serum, and then plated on 25 mm coverslips coated with 100 μg/ml poly-L-lysine (P2636; Sigma-Aldrich, St. Louis, MO, United States) at a density of approximately 325,000 cells per 35 mm dish. Hippocampal neurons were cultured in Neurobasal medium (21103049; Gibco/Thermo Fisher Scientific, Waltham, MA, United States) supplemented with B-27 (17504044; Gibco/Thermo Fisher Scientific, Waltham, MA, United States), penicillin/streptomycin (30002CI; Thermo Fisher Scientific, Waltham, MA, United States), and GlutaMax (35050061; Gibco/Thermo Fisher Scientific, Waltham, MA, United States), and fed 1/2 volume of fresh growth medium every 7 days post-plating. CalPhos calcium phosphate transfection reagent (631312; Takara, Kusatsu, Shiga, Japan) was used to transfect neurons on days *in vitro* (DIV) 10. Animal care and use was conducted following National Institutes of Health guidelines, and procedures were approved by the Institutional Animal Care and Use Committee at Emory University. Cath.-a-differentiated (CAD) cells (Qi et al., 1997) were cultured in DMEM/F12 (10-092-CV; Corning, Corning, NY, United States) media supplemented with 8% fetal bovine serum (Atlanta Biologicals, Flowery Branch, GA, United States) and 1% Penicillin/Streptomycin (Invitrogen, Waltham, MA, United States).

### Immunocytochemistry

Hippocampal neurons were fixed for 15 min at room temperature using freshly prepared 4% paraformaldehyde and 4% sucrose in phosphate-buffered saline (PBS). After fixation, neurons were washed three times in PBS, permeabilized in PBS containing 0.3% Triton X-100 for 15 min, and then blocked in PBS supplemented with 1% bovine serum albumin (BSA) and 5% normal goat serum for 1 h, all at room temperature. Then, neurons were incubated with primary antibodies in PBS for 1 h at room temperature or overnight at 4°C. Coverslips were washed three times in PBS, and incubated with fluorescent secondary antibodies (Alexa Fluor-488 or Alexa Fluor-546; 1:750; ThermoFisher Scientific, Waltham, MA, United States) in PBS for 1 h at room temperature. Coverslips were washed three times in PBS, and then mounted on slides using Fluoromount-G (Southern Biotech, Birmingham, AL, United States). Imaging was carried out using a Nikon C2 laser-scanning confocal system with an inverted Nikon Ti2 microscope (60× Plan Apo objective, 1.4 NA) and a 3× digital zoom. To image spines, Z-stacks comprised of 16–21 optical sections (0.2 μm steps) were acquired, and 2D images were generated from maximum intensity projections.

### Immunoprecipitations and Far-western blotting

Immunoprecipitations were performed by lysing hippocampal tissue, cultured hippocampal neurons, or CAD cells in non-denaturing lysis buffer [50 mM Tris–HCl (pH 7.4), 150 mM NaCl and 0.5% NP-40, complete mini protease inhibitor tablet (Roche, Basel, Switzerland)]. Lysates were spun at 13,000 rpm for 10 min at 4°C. Protein A-magnetic beads (Dynabeads; Invitrogen, Waltham, MA, United States) were bound to appropriate antibodies in PBS-tween for 10 min at room temperature. The lysate supernatant was added to antibody-bound magnetic beads and incubated for 10 min at room temperature. Samples were loaded onto 4–20% Tris-glycine gradient gels (Bio-Rad, Hercules, CA, United States) and transferred to nitrocellulose membranes for blotting. For Far-western blotting we followed the protocol of Wu et al. (2007). Briefly, samples were transferred to PVDF membranes, denatured and renatured using a dilution series of guanidine-HCl, incubated with one μg/ml of purified recombinant CP, and CP was then detected by blotting for 6x-His tag.

### Live-cell imaging

Hippocampal neurons were cultured and imaged in phenol-red free Neurobasal medium supplemented with B-27, GlutaMax, and penicillin/streptomycin. Coverslips were placed in live-cell imaging chambers, and imaged using a Nikon C2 laser-scanning confocal system with an inverted Nikon Ti2 microscope (60× Plan Apo objective, 1.4 NA). Z-stacks comprised of 9 optical sections (0.3 μm steps) were acquired every 30 s for 30 min. Maximum intensity projections were generated and time-lapse images were analyzed using the manual tracking plugin, ImageJ software national institutes of health (NIH).

### Image analysis

ImageJ software was used to visualize and measure spine density and morphology. Samples were blinded before image analysis. Measurements of dendritic protrusions from fixed cells were averaged from two dendritic segments per neuron, each segment coming from different secondary or tertiary branches at least 30 μm in length. We defined filopodial-like protrusions as those having a length greater than or equal to 2 μm, with no discernable spine head. Spines with head protrusions were classified by the presence of at least one thin filopodial extension (≥0.2 μm) emanating from a bulbous head. Temporally color-coded images of dendritic protrusion dynamics were generated in ImageJ following the method of Sanchez-Arias et al. (2020). Nikon Elements software was used to analyze spine head/shaft ratio, quantify the number of CP + protrusions, and measure mean fluorescence intensity values. GraphPad Prism v.7. was used for statistical analysis of data representing at least three replicates from independently prepared samples. Unless otherwise specified, data are presented as the mean ± SEM, with in-text values stated.

## Results

### Capping protein is a positive regulator of dendritic protrusion stability, with implications for the filopodia to spine transition

**FIGURE 1:**
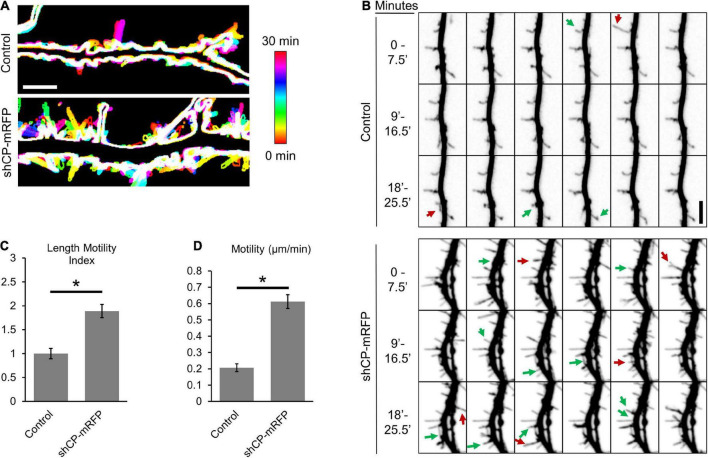
Capping protein (CP) stabilizes dendritic protrusions. **(A)** Representative color-coded images show increased protrusion dynamics in CP knockdown neurons (co-expressing mRFP) compared to control neurons (mRFP). 30-min time-lapse recordings of protrusions were acquired from secondary or tertiary dendritic branches from DIV15 hippocampal neurons every 30 s. Maximum-intensity z-projections were created, thresholded to generate cell outlines, and temporally color-coded to allow visualization of protrusion dynamics. Scale bar, 5 μm. **(B)** CP knockdown reduces the stability of dendritic protrusions. Montage of representative time-lapse video highlighting growing (green arrows) and retracting (red arrows) dendritic protrusions. 30-min time-lapse of DIV15 hippocampal neurons, transfected at DIV10 with either mRFP (Control) or shCP-mRFP. Quantification shows significantly increased rates of Length Motility Index (LMI) **(C)** and Motility **(D)** in CP knockdown neurons (*N* = 5 neurons; 8–11 protrusions/neuron). Scale bar, 5 μm. Error bars represent SEM. *P < 0.0001; Mann–Whitney test.

Dendritic filopodia and immature spines are dynamic structures that repeatedly extend and retract as they search out presynaptic contacts. However, the formation of functional synapses requires the stabilization and maturation of spines. Our previous study suggested that CP functions in the transition of filopodia into spines (Fan et al., 2011). Therefore, we wondered whether CP is involved in spine stability. To determine this, we performed live imaging of dendritic protrusions from cultured hippocampal neurons depleted of CP. At DIV10, we transfected hippocampal neurons with either soluble mRFP (control) or mRFP plus an shRNA targeting CP (shCP-mRFP) (Mejillano et al., 2004; Fan et al., 2011). Then, dendritic protrusions were imaged at DIV15 when filopodia-like protrusions are still relatively abundant in both our control and CP knockdown cultures. In control neurons, most of the dendritic protrusions maintained their length throughout the imaging period, with only minor morphological alterations observed (Figure 1A). Of the few protrusions from control cells that did change their length, most only grew or retracted over a relatively small range (Figure 1A). Conversely, we found that protrusions from shCP-mRFP neurons appeared to grow and retract more often, and over much larger distances (Figure 1A). In addition, we found that there were more protrusions in the CP knockdown neurons that either appeared or disappeared within the 30-min imaging period (Figure 1B). To quantify changes in the length of each protrusion, we tracked the tips of individual protrusions at each timepoint. Using the Length Motility Index (LMI) (Konur and Yuste, 2004) to measure the extent of protrusion extension and retraction, we found that CP knockdown neurons were significantly more motile (Figure 1C). Supporting this, we also found that the velocity of protrusion movement was significantly increased in CP knockdown cells (Figure 1D). This suggests that CP is important for the stability of dendritic protrusions.

### Interactions with CPI-motif proteins are essential for capping protein localization and function in spines

**FIGURE 2:**
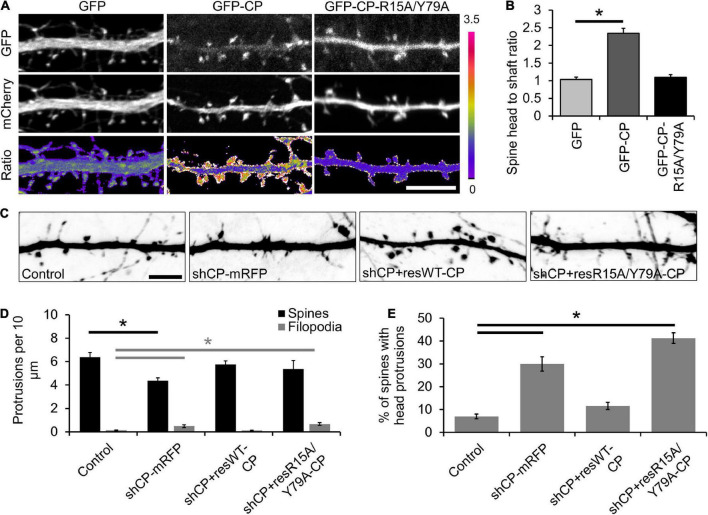
Capping protein interaction (CPI)-motif proteins regulate capping protein (CP) localization and spine morphology. **(A)** Representative images of DIV21 neurons co-expressing mCherry (volume marker) with either green fluorescent protein (GFP), GFP-CP, or GFP-CP-R15A/Y79A. Bottom row, GFP/mCherry ratiometric images. Scale bar, 5 μm. **(B)** CP enrichment in spines requires interactions with CPI-motif proteins. Quantification of spine enrichment, expressed as the ratio of GFP fluorescence intensity in a spine head to the intensity of an equal-size area in the neighboring dendritic shaft. *N* = 18 neurons (12 protrusions/cell). Error bars represent SEM. *P < 0.0001; one-way ANOVA with Tukey’s *post hoc* test. **(C)** CPI-motif interactions are necessary to rescue spine defects in CP knockdown neurons. Representative images of dendritic protrusions from neurons expressing pSuper-mCherry (control), shCP-mRFP, shCP + resWT-CP, and shCP + resR15A/Y79A-CP. Scale bar, 5 μm. **(D)** Quantitative analysis of filopodia and spine density. **(E)** Analysis of the percentage of spines containing spine head protrusions. Error bars represent SEM. *P < 0.01; one-way ANOVA with Tukey’s *post hoc* test.

CPI-motif proteins regulate the proper localization and function of CP in non-neuronal cells (Edwards et al., 2015). To better understand how CP is localized to spines, we utilized a CPβ mutant (R15A/Y79A) that is defective in binding to CPI-motif proteins (Edwards et al., 2015). We co-expressed GFP-CP or GFP-CP-R15A/Y79A along with mCherry (as a soluble marker) at low levels in cultured hippocampal neurons. Unlike GFP-CP, we found that the R15A/Y79A mutant is diffusely distributed throughout dendrites with no obvious enrichment in spines (Figure 2A). To quantify spine enrichment, we measured the ratio of GFP fluorescence in individual spine heads compared with the neighboring dendritic shaft (H/S ratio). The H/S ratio of GFP-CP is significantly higher than GFP alone, while GFP-CP-R15A/Y79A is indistinguishable from GFP (Figure 2B). This suggests that CPI-motif proteins are essential for CP enrichment in dendritic spines.

Next, we sought to determine whether CPI-motif proteins are important for CP function during spine development by knocking down endogenous CP and rescuing its expression using either shRNA-resistant GFP-CP-wildtype (shCP + resWT-CP) or GFP-CP-R15A/Y79A (shCP + resR15A/Y79A-CP) (Figure 2C). We found that CP knockdown increased the density of filopodia-like protrusions, and decreased the density of spines (Figure 2D). We also observed a significant increase in the percentage of spines with protrusions extending from their bulbous heads (spine head protrusions) (Figure 2E). Importantly, while these defects could be rescued by expression of wild-type GFP-CP (shCP + resWT-CP), the GFP-CP-R15A/Y79A mutant (shCP + resR15A/Y79A-CP) was unable to restore normal spine morphology (Figures 2C–E). This suggests that interactions with CPI-motif proteins are required for both CP localization and function during spine development.

### Twinfilin-1 knockdown increases protrusion instability, phenocopying capping protein loss of function

**FIGURE 3:**
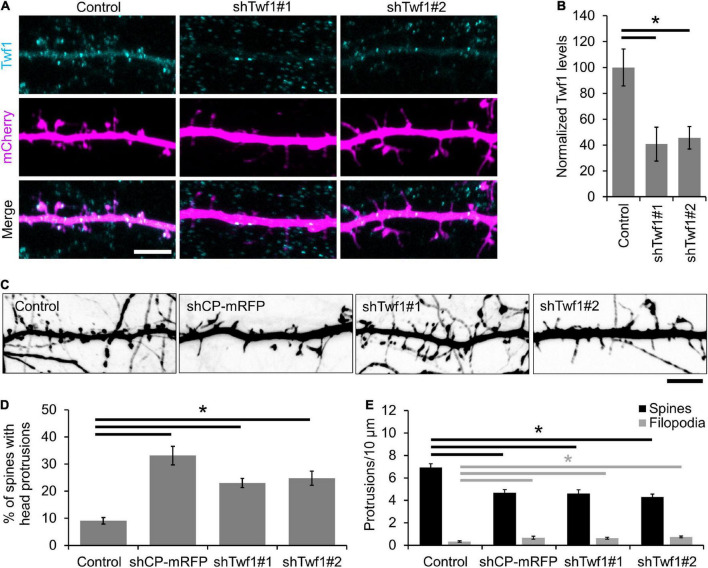
Twinfilin-1 (Twf1) knockdown decreases spine density and increases filopodia density. **(A)** Representative images of control, shTwf1#1, or shTwf1#2 DIV21 hippocampal neurons expressing mCherry (magenta) and immunostained for endogenous Twf1 (cyan). Scale bar, 5 μm. **(B)** Mean Twf1 fluorescence intensity relative to control cells. **(C)** Twf1 knockdown phenocopies capping protein (CP) knockdown in spines. Representative images of dendritic protrusions from neurons expressing shCP-mRFP, shTwf1#1, and shTwf1#2. Scale bar, 5 μm. **(D)** Analysis showing the percentage of spines containing spine head protrusions. **(E)** Analysis of filopodia and spine density. *N* = 18 neurons for control, 10 neurons for shCP-mRFP, 16 neurons for Twf1#1, 19 neurons for Twf1#2. Error bars represent SEM. *P < 0.05; one-way ANOVA with Tukey’s *post hoc* test.

A recent study by Spence et al. (2019) revealed a role for the CPI-motif protein CARMIL3 in spine development. However, the loss of CARMIL3 had only a partial effect on the localization of CP to spines (Spence et al., 2019), suggesting that multiple CPI-motif proteins regulate the localization and function of CP in spines. To test this, we knocked down the CPI-motif-containing protein Twf1 by generating two separate shRNAs (shTwf1#1 and shTwf1#2) against unique, non-overlapping sequences. We confirmed Twf1 knockdown by immunostaining for endogenous Twf1 in DIV21 hippocampal neurons expressing the shRNAs (Figure 3A). In control cells, Twf1 can be found in both dendritic spines and the dendritic shaft. However, the expression of shTwf1#1 and shTwf1#2 resulted in a 59.2 and 54.3% decrease in overall Twf1 fluorescence compared to control cells, respectively (Figure 3B). Next, we examined the effects of Twf1 knockdown on spine density and morphology (Figure 3C). We found that Twf1 knockdown leads to a significant increase in the percentage of spines containing head protrusions (Figure 3D), similar to CP knockdown neurons as shown in Figure 2E. Likewise, we also detected a decrease in spine density, and an increase in filopodia density in Twf1 knockdown cells relative to controls (Figure 3E). This suggests that Twf1 is required for normal spine morphogenesis.

**FIGURE 4:**
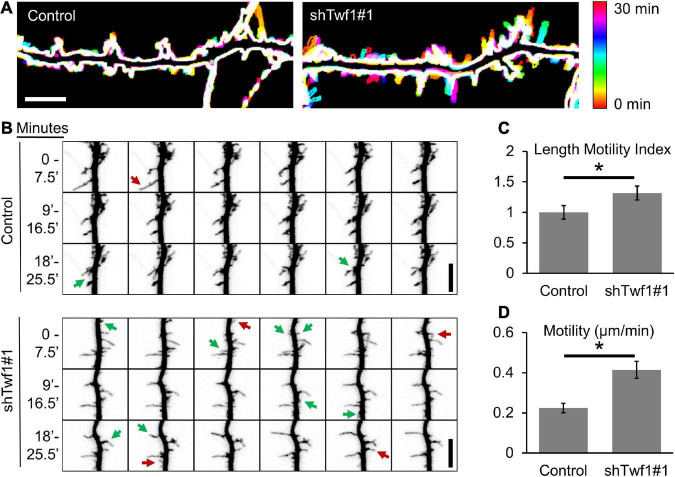
Twinfilin-1 (Twf1) knockdown destabilizes dendritic protrusions. **(A)** 30-min time-lapse of DIV15 hippocampal neurons, transfected at DIV11 with either pSuper-mCherry (Control) or shTwf1#1 (which co-expresses mCherry). Temporally color-coded images show increased protrusion dynamics in Twf1 knockdown neurons relative to controls. **(B)** Montage of a time-lapse video highlighting growing (green arrows) and retracting (red arrows) dendritic protrusions. Quantification shows significantly increased rates of Length Motility Index (LMI) **(C)** and Motility **(D)** in Twf1 knockdown neurons (*N* = 5 neurons; 8–15 protrusions/neuron). Scale bars, 5 μm. Error bars represent SEM. *P < 0.05; Mann–Whitney test.

To determine whether the loss of Twf1 affects spine stability similar to CP knockdown, we imaged dendritic protrusions in live neurons at DIV15 (Figure 4A). Indeed, we found that protrusions from Twf1 knockdown neurons were more dynamic than protrusions from control cells (Figure 4B), suggesting that Twf1 is important for the stability of dendritic protrusions. We detected small but significant increases in both LMI and protrusion tip velocity of dendritic protrusions from Twf1 knockdowns, compared to controls (Figures 4C,D). These data suggest that Twf1 is likely to function in the same pathway as CP in regulating actin-based spine development.

### Twinfilin-1 localization to spines requires actin-binding

**FIGURE 5:**
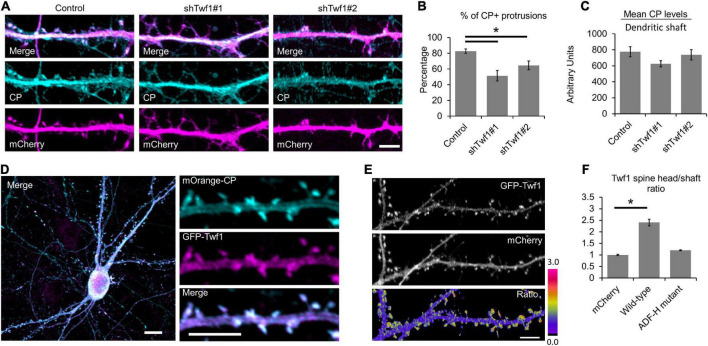
Twinfilin-1 (Twf1) is enriched in spines. **(A)** Knockdown of Twf1 reduces capping protein (CP) enrichment in dendritic protrusions. Representative images of DIV21 hippocampal control, shTwf1#1, and shTwf1#2 neurons co-expressing mCherry (magenta), fixed and immunostained for endogenous CP (cyan). Scale bar, 5 μm. **(B)** Analysis shows the percentage of dendritic protrusions that contain CP. *N* = 19 neurons. **(C)** Analysis of mean CP fluorescence intensity relative to control. *N* = 19 neurons. **(D)** Representative image of DIV21 hippocampal neuron co-expressing mOrange-CP (cyan) and GFP-Twf1 (magenta). Inset shows magnified view of CP and Twf1 localization in spines. Scale bars: main image = 10 μm, inset = 5 μm. **(E)** Representative images of GFP-Twf1, mCherry, and their ratiometric pseudocolor presentation highlighting the Twf1 spine enrichment. Scare bar = 5 μm. **(F)** Quantification of spine head enrichment relative to the neighboring dendritic shaft for wild-type Twf1 and the actin depolymerization factor homology (ADF-H) domain mutant of Twf1 compared to soluble mCherry. *N* = 16 neurons (12 protrusions/cell) per condition. Error bars represent SEM. *P < 0.05; one-way ANOVA with Tukey’s *post hoc* test.

Because the knockdown of Twf1 induces a spine phenotype similar to CP knockdown, we wondered whether Twf1 affects CP localization. To test this, we knocked down Twf1 and immunostained for endogenous CP (Figure 5A). Our analysis revealed a significant reduction in the percentage of protrusions containing CP in Twf1 knockdown cells (Figure 5B). However, the total levels of CP in the dendrites of Twf1 knockdown neurons were similar to control (Figure 5C). This suggests that Twf1 regulates the targeting of CP to spines, likely along with additional CPI-motif containing proteins. Next, we confirmed that Twf1 is itself enriched in dendritic spines, and that Twf1 colocalizes with CP, by co-expressing mOrange-CP and GFP-Twf1 in DIV21 neurons (Figure 5D). The spine enrichment of Twf1 is better depicted by the ratiometric projection of GFP-Twf1 and mCherry (Figure 5E). By measuring the H/S ratio, we further confirmed that GFP-Twf1 is enriched in spines, unlike soluble mCherry (Figure 5F). We also examined the localization of a Twf1 mutant that is defective in binding to actin (ADF-H-domain mutant, R96A/K98A/R267A/R269A) (Hakala et al., 2021). We found that the actin depolymerization factor homology (ADF-H)-domain mutant of Twf1 was diffusely distributed throughout dendrites and was essentially indistinguishable from soluble mCherry (Figure 5F). This suggests that the proper localization of Twf1 in spines depends upon its actin-binding activity.

### Capping protein interacts with Shank to regulate postsynaptic development

**FIGURE 6:**
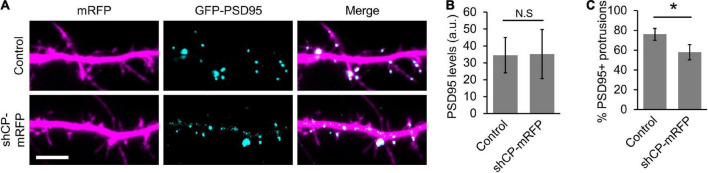
Capping protein (CP) knockdown negatively affects PSD95 clustering in spines. **(A)** Representative dendritic regions showing that CP knockdown decreases the number of protrusions containing GFP-PSD95. **(B)** Quantification shows no significant difference in the levels of GFP-PSD95 between control and shCP-mRFP cells. **(C)** Quantification shows significant reduction in the percentage of dendritic protrusions containing GFP-PSD95 puncta. *N* = 19 cells per condition. Scale bar, 5 μm. Error bars represent SEM. *P < 0.001; Mann–Whitney test.

The actin cytoskeleton also plays an important role in the development and modification of the PSD. Not only is the development of the PSD coincident with spine head formation (Arellano et al., 2007; De Roo et al., 2008), but the PSD is also intimately connected to F-actin networks through scaffold proteins (Qualmann et al., 2004; Carlisle and Kennedy, 2005; Kuriu et al., 2006; Zeng et al., 2018). The sequential recruitment and accumulation of membrane associated guanylate kinase (MAGUK) proteins, such as PSD95, has previously been proposed to stabilize newly formed spines (Marrs et al., 2001; Prange and Murphy, 2001; Lambert et al., 2017). Therefore, we wanted to determine whether PSD95 clustering is affected in CP knockdown neurons. We co-transfected hippocampal neurons with low levels of GFP-tagged PSD95 and either soluble mRFP (control) or mRFP plus an shRNA targeting CP (shCP-mRFP) (Mejillano et al., 2004; Fan et al., 2011). We then visualized the clustering of GFP-PSD95 in dendritic protrusions at DIV16, when a majority of the protrusions were transitioning from filopodia to spines. Overall, the total levels of GFP-PSD95 were similar in both control and CP knockdown cells (Figures 6A,B). However, we found that there were significantly less protrusions containing GFP-PSD95 puncta in shCP-mRFP cells, as compared to control cells (Figures 6A,C). This suggests that CP is involved in the accumulation of postsynaptic components to spines during development.

**FIGURE 7:**
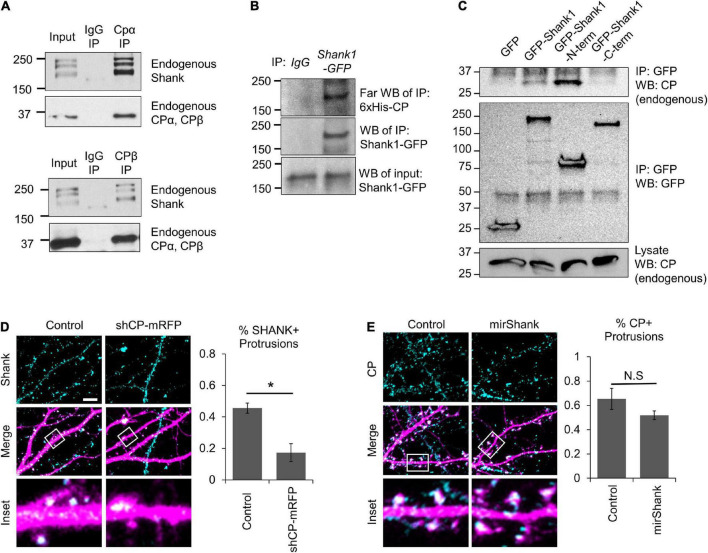
Capping protein (CP) association with Shank scaffolding proteins. **(A)** Co-IP of endogenous Shank proteins with endogenous CP from isolated adult rat hippocampi using two separate antibodies, against either CPα or CPβ. **(B)** Far-western blots showing that purified recombinant 6xHis-CP directly associates with Shank1-GFP from Cath.-a-differentiated (CAD) cell α-GFP immunoprecipitation (IP). **(C)** Endogenous CP co-IPs with GFP-Shank1 from CAD cell lysates. A *N*-terminal fragment of Shank1 (containing the SPN domain, Ankyrin repeats, and SH3 domain) associates with CP, while the C-terminal fragment (containing the PDZ domain, Proline-rich motifs, and SAM domain) does not. **(D)** Left, representative images of DIV21 hippocampal neurons expressing mRFP (control) or shCP-mRFP (magenta) and stained for endogenous Shank (cyan). Right, CP knockdown significantly reduced the percentage of spines containing Shank puncta, as compared to control cells. *N* = 14 cells per condition. **(E)** Left, representative images of DIV21 hippocampal neurons expressing mirScramble-GFP (control) or mirShank-GFP (magenta) and stained for endogenous CP (cyan). Right, Quantification shows no effect of Shank knockdown on the percentage of spines containing CP puncta, relative to control neurons. *N* = 18 cells per condition. Scale bar, 5 μm. Error bars represent SEM. *P < 0.05; Mann–Whitney test.

We wondered whether the clustering of GFP-PSD95 by CP required interactions with any specific components of the PSD. We performed a preliminary immunoprecipitation (IP) of CP and mass-spectrometry analysis, and identified Shank scaffolding proteins as putative CP interactors. We confirmed the interaction between CP and Shank by co-IP of endogenous Shank with endogenous CP (Figure 7A). This corroborates a previous proteomic screen that identified CPβ as a putative GFP-Shank3 interactor (Peca et al., 2011). We next wanted to determine whether CP and Shank interact directly, so we performed a Far-western blot analysis (Wu et al., 2007). Here, Shank1-GFP was expressed in CAD cells, and immunoprecipitated using anti-GFP antibody, followed by incubation with purified 6xHis-CPα1/β2 and blotting. We detected a robust interaction between purified recombinant 6xHis-CP and Shank1-GFP by Far-western blotting, indicating that CP and Shank1 interact directly (Figure 7B). To determine which domains of Shank1 interact with CP, we expressed the *N*-terminal and *C*-terminal regions of GFP-Shank1 and assayed for the co-precipitation of endogenous CP. Our data suggest that the CP-Shank interaction site resides within the *N*-terminus of Shank1 (Figure 7C).

Next, we analyzed the effects of CP knockdown on the distribution of Shank in hippocampal neurons. We found that CP depletion significantly reduced the percentage of dendritic protrusions containing Shank puncta (Figure 7D), suggesting that CP is important for Shank enrichment. Interestingly, Shank is known to regulate actin cytoskeleton remodeling during spine development through interactions with other actin-binding proteins including ABI, cortactin, and the WAVE complex (Wang et al., 2020). Therefore, we wanted to know whether Shank affects CP localization to spines. To test this, we simultaneously knocked down all three Shank family members using a miRNA strategy (MacGillavry et al., 2016) and examined endogenous CP signals in spines. We found that Shank knockdown had no significant effects on the percentage of protrusions containing CP (Figure 7E), suggesting that CP localization to spines does not require Shank proteins. Instead, our results suggest that CP is involved in the clustering of postsynaptic components in spines, which can further stabilize dendritic protrusions leading to spine maturation.

## Discussion

We have revealed a role for CP in dendritic spine stabilization and morphological development. The localization of CP to spines, and its role in maintaining proper spine morphology, depends upon interactions with CPI motif-containing proteins. This finding is not entirely surprising, given that in non-neuronal cells CP localization to the leading edge requires interactions with CPI-motif containing proteins (Edwards et al., 2015). Other studies in non-neuronal cells have shown that CP is largely regulated by CARMIL, Twf, and V-1/myotrophin (Taoka et al., 2003; Falck et al., 2004; Bhattacharya et al., 2006; Stark et al., 2017). Most of the total cellular population of CP is inactive, and bound to V-1 (Fujiwara et al., 2014). V-1 binding to CP inhibits CP association with the plus end and renders CP cytosolic, thus providing a diffusible pool of inactive CP throughout the cell (McMillen and Vavylonis, 2016). CARMIL2 and CARMIL3 are expressed in the brain, where they can bind to CP and regulate actin assembly at the plasma membrane (Stark et al., 2017). CARMIL proteins can allosterically disengage CP from V-1, thereby activating CP for the barbed end capping of actin filaments. Importantly, the CARMIL-CP interaction also allosterically weakens the CP/barbed end interaction, which can lead to CP dissociation from the barbed end and rapid turnover. However, recent work indicates that plus end capping is enhanced when Twf (Paavilainen et al., 2004) binds to CP at an overlapping region to compete CARMILs off CP, resulting in a “pro-capping” effect (Johnston et al., 2018). A separate study recently demonstrated that CARMIL3 regulates the structural and functional transition of dendritic filopodia to mature spines in cultured hippocampal neurons (Spence et al., 2019). However, they found that depletion of CARMIL3 resulted in a small but significant loss of CP from spines, suggesting that CP localization in spines is regulated by additional binding proteins.

Here, we show that Twf1 knockdown results in a similar partial loss of CP in spines, indicating that CP enrichment in spines depends on Twf1. In budding yeast, CP was shown to be essential for the proper localization of Twf to actin patches (Palmgren et al., 2001). However, our data shows that a Twf1 mutant defective in actin binding (ADF-H domain mutant) does not localize properly to dendritic spines. This suggests that Twf1 enrichment in spines does not depend on CP, as the CPI-motif binding of Twf1 resides in its *C*-terminal tail region (Johnston et al., 2018; Takeda et al., 2021). Previous work has also shown that Twf readily associates with actin filament barbed ends either alone or as part of a complex with CP (Palmgren et al., 2001; Paavilainen et al., 2007; Johnston et al., 2018). In either case, Twf1 is able to halt further actin filament elongation (Paavilainen et al., 2007; Hilton et al., 2018). When found in complex with CP on barbed ends, Twf1 catalyzes the dissociation of CP from barbed ends (Hakala et al., 2021). In cooperation with V-1, CP dissociates from Twf1 leaving Twf1 alone at the barbed end to regulate actin filament depolymerization (Johnston et al., 2015; Shekhar et al., 2021). As a result, Twf1 is considered to be an anti-capping and depolymerizing factor (Hakala et al., 2021; Mwangangi et al., 2021; Shekhar et al., 2021). However, our finding that Twf1 knockdown appears to phenocopy CP knockdown is consistent with the “pro-capping” effect of Twf1 reported recently (Johnston et al., 2018). It is likely that specific effects of Twf1 on barbed end capping may be context specific and depend on the local availability of other actin-binding and CP regulatory proteins. Nonetheless, interactions with Twf and CARMIL represent an important mechanism for dynamically regulating CP activity and its association with actin filament barbed ends in spines. More generally, the activities of various CPI motif-containing proteins may be temporally and spatially regulated during both development and synaptic plasticity to control barbed end capping. Considering that the CPI motif-binding-deficient CP mutant does not localize to spines at all, it is likely that other CPI motif-containing proteins (i.e., CD2AP/CIN85, CKIP-1, FAM21, CAPZIP, etc.) are also involved in CP regulation and localization.

In this study we found that CP promotes the stabilization of dendritic protrusions, and the recruitment of important scaffolding proteins such as Shank and PSD95 to nascent spines. The stabilization of spines is a crucial step during neuronal development, and it is essential for long-term memory formation (Koleske, 2013). For example, training in a novel motor skill induces the rapid formation of dendritic spines in the mouse motor cortex (Xu et al., 2009). Yet, the long-term maintenance of these motor memories requires the preferential stabilization of nascent spines through subsequent training (Xu et al., 2009). This was confirmed using an elegant optogenetics-based approach to directly shrink nascent spines following motor learning, leading to memory erasure (Hayashi-Takagi et al., 2015). However, despite the importance of spine stabilization for memory formation, many of the underlying mechanisms remain unclear. Previous studies have identified several signaling pathways that can contribute to the stabilization of spines, including the accumulation of postsynaptic MAGUK scaffolding proteins, activity-dependent calcium transients, EphB receptor tyrosine activity, BDNF-stimulation, and integrin-mediated signaling (Okabe, 2020). Ultimately however, these pathways converge on the actin cytoskeleton and its associated regulatory proteins.

Capping protein’s function in spine stabilization could be due to its role in spine head expansion and/or the subsequent accumulation of MAGUKs and AMPARs (α-amino-3-hydroxy-5-methyl-4-isoxazolepropionic acid receptors) through the recruitment of Shank proteins. Expansion of the spine head requires the formation of a highly branched actin network at the distal end of dendritic filopodia *via* the cooperative activity of CP and Arp2/3 (Falet et al., 2002; Spence and Soderling, 2015). In this instance, increased capping activity essentially halts the polymerization of existing actin filament barbed ends, thereby causing most of the actin polymerization to occur on newly formed Arp2/3-dependent branched filaments. It has been proposed that the expanded spine head is then able to support the sequential recruitment and accumulation of MAGUK proteins that gradually stabilizes nascent spines and facilitates the formation of a functional postsynaptic structure (Lambert et al., 2017). This model is supported by our finding that CP knockdown decreases the recruitment of PSD95 to spines and leads to instability. It is also supported by another study showing that Arp2/3 is necessary for AMPA receptor recruitment to spines, though interestingly Arp2/3 is not required for MAGUK recruitment (Spence et al., 2016).

We propose that the capping of actin filaments and the subsequent formation of an enlarged spine head is a prerequisite for the CP-dependent recruitment of Shank and MAGUK proteins to spines. In non-neuronal cells, the knockdown of CP increases the number of filopodia and alters filopodia morphology (Sinnar et al., 2014), similar to our observations of dendritic protrusions in neurons. However, CP knockdown in epithelial cells increases the proportion of uniformly shaped and “cattail”-shaped filopodia, while decreasing the proportion of filopodia with a tapered-shape (Sinnar et al., 2014). In addition, CP depletion in non-neuronal cells reduces filopodial dynamics (Sinnar et al., 2014). While these results appear somewhat contradictory to our findings, it is important to note that conventional filopodia differ from dendritic filopodia in numerous ways structurally, functionally, and molecularly. For example, actin filaments within conventional filopodia are uniformly oriented with their barbed ends facing the filopodial tip and tightly crosslinked into linear bundles by Fascin (Okabe, 2020). On the other hand, dendritic filopodia contain a loose network of actin filaments of varying length and mixed polarity, containing a mix of both actin bundles and branched filaments (Korobova and Svitkina, 2010). Similarly, some actin binding proteins such as Fascin and Myosin-X are found only in conventional but not dendritic filopodia, while others such as Myosin II are only found in dendritic filopodia (Korobova and Svitkina, 2010). Moreover, while CP itself has been found in conventional filopodia, it is not nearly as abundant there as it is in dendritic protrusions. Altogether, this suggests that the function of CP is very different in these two structures.

Capping of the barbed end of actin filaments represents an important mechanism to regulate filament length and stability (Pollard and Borisy, 2003). CP is ubiquitously expressed, and is the predominant plus-end actin capper. We previously reported that CP knockdown impairs spine development and synapse formation, resulting in a reduction in spines with expanded heads, an increase in dendritic filopodia, and the formation of aberrant filopodia-like protrusions from existing spine heads (Fan et al., 2011). CP likely also plays a role in spine remodeling during synaptic potentiation (Kitanishi et al., 2010; Kuboyama et al., 2020), although this has not been investigated. It should be noted that epidermal growth factor receptor pathway substrate 8 (Eps8) has also been shown to cap the (+) end and to regulate spine formation and plasticity (Menna et al., 2013; Stamatakou et al., 2013). The findings that knockdown either CP or Eps8 resulted in similar spine deficits suggest that one cannot compensate for the loss of the other. How Eps8 and CP function differently in spine development remains unknown. In any case, capping of barbed ends of actin filaments in spines appear to be essential for not only the spine structure but also for the protein complexes underlying postsynaptic function. In addition, capping of the minus ends of actin filaments by Tropomodulin family members also regulates spine development and dendritic arborization (Yamashiro et al., 2012; Omotade et al., 2018). Therefore, capping of both ends of actin filaments represents an important mechanism for spine formation and synapse development. Although how the activity of all these capping proteins is coordinated during spine development and synaptic plasticity remains unknown.

The SH3 and multiple ankyrin repeat domains (Shank) family of proteins are “master organizers” of the PSD (Sheng and Kim, 2000; Monteiro and Feng, 2017; Kursula, 2019). Deletion of Shank has been shown to reduce the PSD levels of several proteins including Homer, SAPAP (synapse-associated protein 90/PSD95-associated protein), NMDARs (N-methyl-D-aspartate receptors) and AMPARs (Won et al., 2012; Han et al., 2013). The loss of Shank also leads to a reduction in spine number and size (Sala et al., 2001; Roussignol et al., 2005; Mei et al., 2016; Zhou et al., 2016), as well as a decrease in AMPAR transmission (with observed effects on the frequency and amplitude of miniature excitatory postsynaptic currents) (Peca et al., 2011; Mei et al., 2016; Peixoto et al., 2016; Wang et al., 2016; Zhou et al., 2016). Moreover, mutations in Shank have been associated with a number of neuropsychiatric and neurodevelopmental disorders (Kursula, 2019). A recent study has also identified an autism-linked missense mutation in SHANK3 that causes defects in Shank3 recruiting a number of actin-binding proteins to the PSD, leading to the disruption of spine development (Wang et al., 2020). How are Shank proteins targeted to nascent dendritic protrusions during postsynaptic development? Cortactin is an F-actin and Arp2/3 binding protein concentrated in dendritic spines. Cortactin binds directly to Shank, F-actin, and Arp2/3; it may coordinate actin-based spine formation and Shank-mediated PSD assembly. Previous studies suggest that Shank likely acts upstream of Cortactin to promote the establishment and modification of the actin-based spine structure during synapse formation and plasticity (Zeng et al., 2018). A proteomic analysis using GFP-Shank3 transgenic mouse identified over 270 proteins that may interact with Shank3. Although CPβ was one of those proteins immunoprecipitated from the synaptosomal fraction, their binding was not confirmed and the nature of this interaction was not further investigated (Durand et al., 2012). Furthermore, Twf1 has previously been identified as a part of the interactome of Shank3 (Wang et al., 2020). Therefore, Twf1 regulated CP localization to nascent dendritic spines likely plays a role in F-actin remodeling during spine development and maturation, as well as in the recruitment and accumulation of Shank proteins underlying the PSD development. Future studies utilizing biochemical, cell biology, and neuroscience approaches are needed to fully elucidate the CP-Shank interaction, its function in synapse development and plasticity, and how its disruption may contribute to synaptic dysfunction and neurological disorders.

In conclusion, we have provided evidence that CP is important for spine development and stability, as well as the assembly of postsynaptic apparatus, largely through the activity of CPI motif-containing proteins. CP has been implicated in a number of brain disorders including Down syndrome (Gulesserian et al., 2002), dementia (Kitanishi et al., 2010), and Intellectual Disability (Huang et al., 2020), highlighting the importance of CP in normal brain function and its disruption in brain disorders. Moreover, the relatively high levels of CP in postnatal hippocampi (Fan et al., 2011) supports an important role for CP in postnatal brain development and function. Future studies will aim to identify how CPI motif-containing proteins coordinate CP activity, and determine how various actin cappers cooperate during development and plasticity.

## Data availability statement

The raw data supporting the conclusions of this article will be made available by the authors, without undue reservation.

## Ethics statement

The animal study was reviewed and approved by the Institutional Animal Care and Use Committee at Emory.

## Author contributions

KM designed and performed most of the experiments. YF performed the initial proteomic screening and co-IP. PM and JC helped with the CP-Shank interaction experiments. JZ and KM oversaw the project and designed the research. All authors contributed to the article and approved the submitted version.
